# Differential Regulation of GABA_B_ Receptor Trafficking by Different Modes of *N*-methyl-d-aspartate (NMDA) Receptor Signaling*

**DOI:** 10.1074/jbc.M113.487348

**Published:** 2014-01-14

**Authors:** Sriharsha Kantamneni, Immaculada M. Gonzàlez-Gonzàlez, Jia Luo, Helena Cimarosti, Susan C. Jacobs, Nadia Jaafari, Jeremy M. Henley

**Affiliations:** From the School of Biochemistry, Medical Sciences Building, University of Bristol, Bristol BS8 1TD, United Kingdom

**Keywords:** G Protein-coupled Receptors (GPCR), GABA Receptors, Glutamate Receptor Ionotropic (AMPA, NMDA), Neurodegeneration, Neurotransmitter Receptors, Receptor Endocytosis, Receptor Recycling, GABAB Receptor, Chem-LTP, Oxygen-glucose Deprivation (OGD)

## Abstract

Inhibitory GABA_B_ receptors (GABA_B_Rs) can down-regulate most excitatory synapses in the CNS by reducing postsynaptic excitability. Functional GABA_B_Rs are heterodimers of GABA_B1_ and GABA_B2_ subunits and here we show that the trafficking and surface expression of GABA_B_Rs is differentially regulated by synaptic or pathophysiological activation of NMDA receptors (NMDARs). Activation of synaptic NMDARs using a chemLTP protocol increases GABA_B_R recycling and surface expression. In contrast, excitotoxic global activation of synaptic and extrasynaptic NMDARs by bath application of NMDA causes the loss of surface GABA_B_Rs. Intriguingly, exposing neurons to extreme metabolic stress using oxygen/glucose deprivation (OGD) increases GABA_B1_ but decreases GABA_B2_ surface expression. The increase in surface GABA_B1_ involves enhanced recycling and is blocked by the NMDAR antagonist AP5. The decrease in surface GABA_B2_ is also blocked by AP5 and by inhibiting degradation pathways. These results indicate that NMDAR activity is critical in GABA_B_R trafficking and function and that the individual subunits can be separately controlled to regulate neuronal responsiveness and survival.

## Teaser

**Background:** Heterodimerization of GABA_B1_ and GABA_B2_ subunits is required for functional GABA_B_Rs.

**Results:** GABA_B_R subunits are differentially regulated by activation of synaptic or extrasynaptic NMDARs.

**Conclusion:** GABA_B_R trafficking and function is regulated by NMDARs.

**Significance:** GABA_B_Rs are potential targets for treating diseases such as stroke and cerebral ischemia.

## Introduction

γ-Amino butyric acid (GABA),^6^ the main inhibitory neurotransmitter in the mammalian brain, acts at two main classes of receptors, the ionotropic GABA_A_ and metabotropic GABA_B_ receptors. GABA_A_ receptors are fast ligand gated Cl^−^ channels. The GABA_B_ receptor (GABA_B_R) is a heteromeric G-protein-coupled receptor comprising GABA_B1_ and GABA_B2_ subunits that exert much longer lasting synaptic inhibition (1). The GABA_B1_ subunit contains the ligand-binding domain (2), and GABA_B2_ couples to the G-protein to down-regulate adenylate cyclase (3). GABA_B_Rs are present at both post- and presynaptic compartments and changes in their number, activity, and/or localization can dramatically alter the level of synaptic inhibition by activating inwardly rectifying K^+^ channels and inhibiting Ca^2+^ channels (for review, see Refs. 4–7).

The surface expression of most types of G-protein-coupled receptors is down-regulated by agonist-evoked recruitment of G protein-coupled receptor-dependent kinases, β-arrestin binding, endocytosis, and subsequent degradation or recycling (8). However, GABA_B_Rs are atypical. Neither native nor recombinant GABA_B_Rs are G protein-coupled receptor-dependent kinase substrates, and they do not undergo agonist-induced internalization (9, 10). They do, however, display constitutive endocytosis (11), and it has been proposed that they undergo both very rapid endocytic recycling (12, 13) and membrane lateral diffusion (14).

GABA_B_Rs are abundant at glutamatergic synapses (15, 16) where they engage in reciprocal cross-talk with NMDARs (17–20). Sustained glutamate application promotes GABA_B_R endocytosis, sorting to lysosomal degradation and consequent decreased surface expression (10, 21). Prolonged activation of NMDARs results in CaMKII-mediated phosphorylation of GABA_B1_ causing a dynamin- and CaMKII-dependent endocytosis of GABA_B_Rs (22). Additionally, AMP kinase phosphorylation and subsequent protein phosphatase 2A dephosphorylation of GABA_B2_ promotes lysosomal degradation of the endocytosed receptors (23). These studies demonstrate that pharmacological manipulation of NMDARs can influence GABA_B_R surface expression and endosomal trafficking. However, they rely on the sustained bath application of relatively high doses of agonist that will activate all functional NMDARs. This is an important consideration because it is well established that NMDAR activation can be either beneficial or cytotoxic, depending on the location and intensity of stimulation. Activation of synaptic NMDARs is a trigger for synaptic plasticity and can be neuroprotective via nuclear Ca^2+^ signaling, whereas prolonged activation of extrasynaptic NMDARs promotes cell death (24).

Differences in the effects of synaptic and extrasynaptic NMDARs on GABA_B_R trafficking have not been reported. In this study, we tested the hypothesis that different types of NMDAR activation have different affects on GABA_B_R expression and trafficking. We show that selective activation of synaptic receptors using a NMDA receptor-dependent chemically induced LTP protocol (chemLTP) (25, 26) enhances both GABA_B1_ and GABA_B2_ surface expression via increased recycling. In contrast, oxygen/glucose deprivation (OGD), which among other effects, elicits excessive glutamate release and excitotoxic activation of NMDARs (24) and increases GABA_B1_ but decreases GABA_B2_ surface expression. These findings demonstrate that the surface expression of GABA_B_Rs is differentially regulated in response to synaptic (physiological) and global (pathophysiological) stimulation of NMDARs.

## EXPERIMENTAL PROCEDURES

### 

#### 

##### Antibodies and Chemicals

Primary antibodies used were as follows: rabbit anti-GABA_B1a,b_ (Santa Cruz Biotechnology, for Western blotting), guinea pig anti-GABA_B1a,b_ (for all imaging) and anti-GABA_B2_ (Chemicon, Intl., Temecula, CA), and mouse monoclonal anti-β-actin (Sigma-Aldrich). HRP-conjugated secondary antibodies used were goat anti-rabbit IgG, goat anti-mouse IgG, goat anti-rabbit IgG, and goat anti-guinea pig IgG (Sigma-Aldrich). Fluorochrome-conjugated secondary antibodies used were goat anti-rabbit Alexa Fluor 488 (green), goat anti-guinea pig Alexa Fluor 568 (red), and goat anti-mouse Alexa Fluor 568 (red) (Molecular Probes, Eugene, OR). The anti-HA tag (6E2) mouse monoclonal antibody is an Alexa Fluor® 488 conjugate (Cell Signaling).

##### Expression Constructs, Transfection, and Transduction

Cultured hippocampal cells were transfected using Lipofectamine 2000 (Invitrogen). GABA_B_R expression constructs pmyc-GABA_B1a_ and pHA-GABA_B2_ were kind gifts from Steve Moss and Benny Bettler, respectively. Cells were imaged 48 h after transfection. RFP-Rab4 constructs in pSinRep5 and pcDNA vectors were gifts from Jose Esteban. Hippocampal cells were transduced 16 h prior to treatment. For some experiments the RFP tag was changed to a GFP for Rab4 and Rab11. We also made Sindbis containing fluorophore-tagged wild-type and mutant CB1 receptors, which were used as infection and neuronal viability controls for GABA_B_ expression in neurons.

##### Primary Hippocampal/Cortical Neuronal Cultures

Primary hippocampal and cortical neuronal cultures were prepared from embryonic day 18 rats exactly as described previously (27).

##### LTP Protocol

Cultured cortical neurons were washed with LTP buffer (150 mm NaCl, 2 mm CaCl_2_, 5 mm KCl, 10 mm HEPES, 30 mm glucose, 0.5 μm tetradotoxin, 1 μm strychnine, 20 μm bicuculline; pH 7.4) as described previously (25, 26). For the controls, 50 μm AP5 was added 5 min before adding 200 μm glycine. To induce LTP, glycine was added to the cells for 3 min at 37 °C and then replaced with LTP buffer for the times indicated (5, 10, or 20 min).

##### OGD Protocol

On days *in vitro* 14 to 21, the cultures were subjected to OGD exactly as described previously (28). Briefly, neurons were washed twice with OGD medium (1.26 mm CaCl_2_, 5.36 mm KCl, 136.89 mm NaCl, 0.44 mm KH_2_PO_4_, 0.34 mm Na_2_HPO_4_, 0.49 mm MgCl_2_, 0.44 mm MgSO_4_, 25 mm HEPES, 4 mm NaHCO_3_, 1% penicillin/streptomycin; pH 7.2). The medium was then exchanged for OGD medium previously bubbled with N_2_/CO_2_ (95%/5%) for 10 min. The cultures were then transferred to an anaerobic chamber at 37 °C with N_2_-enriched atmosphere, where they were maintained for 30, 45, or 60 min. After OGD, the cells were removed from the chamber, washed twice with PBS, and processed either for biotinylation or imaging. Where appropriate, drugs were incorporated in culture medium and in OGD medium during the indicated periods.

##### Cell-surface Biotinylation

Neurons were biotinylated using the membrane impermeable and cleavable biotinylation reagent sulfosuccinimidyl-2-(biotinamido) ethyl-1,3-dithiopropionate (EZ-Link Sulfo-NHS-SS-biotin) (0.15 mg/ml in PBS, Pierce) for 10 min at 4 °C as described previously (29). The intracellular protein β-actin was used as a control. Bands were quantified using NIH ImageJ software (version 1.30) and normalized to the total receptor fraction. Unpaired Student's *t* tests were performed with a Newman-Keuls post-test for multiple comparison data sets.

##### Endocytosis/Recycling Experiments

GABA_B_R endocytosis and recycling was measured by the decrease of internalized GABA_B_Rs labeled with cleavable (S = S linked) biotin. Cortical cultures were surface biotinylated as described above, and cells were transferred to 37 °C for 30 min to allow endocytosis to occur. Cells were then activated by chemLTP protocol and incubated for the times indicated to allow internalized receptors to recycle back to the surface. The cells were then cooled to 4 °C and incubated with glutathione cleavage buffer (twice for 15 min each at 4 °C) to ensure complete cleavage of surface biotin. Cells were then washed twice with 10 mm iodoacetamide-PBS solution to quench excess glutathione. Residual biotinylated (internalized) receptors were then isolated by streptavidin pull down, and GABA_B_R subunits were detected by Western blotting. The rate of disappearance of biotinylated GABA_B_Rs provides a measure of receptor recycling. Leupeptin was included throughout to block protein degradation.

##### Live Cell Imaging Experiments

Imaging was perfomed using a Zeiss LSM 510 confocal microscope. Dissociated hippocampal neurons were transfected with p*HA*-GABA_B2_ expression vector and used 48 h later. At the beginning of the experiment, HA-reactive sites on the cell surface of neurons expressing HA-GABA_B2_ were labeled with an excess of anti-HA coupled to Alexa Fluor 488 (1:200) at the same time as the glycine/vehicle application (LTP protocol described above). Neurons were quickly washed twice in LTP buffer, and the fluorescence at time zero was acquired. Cells were kept a further 10 min in LTP buffer without glycine and incubated again with HA-Alexa Fluor 488 to label the newly surface inserted HA-GABA_B2_. Neurons were quickly washed twice, and the fluorescence at 10 min was acquired. The same process was repeated for 20 min. Green fluorescence at 0, 10, and 20 min were recorded in the same cell as a series of Z stacks (0.25-μm spacing between single confocal slices). The rate of receptors recycled or exocytosed in an individual cell was then determined from the zero control (*t* = 0) conditions in the same cell as an increase in the fluorescence after 10 and 20 min. Differences in expression were normalized to the mean of the fluorescence at time zero. Statistical analysis of differences between experimental groups was performed using one-way analysis of variance followed by post hoc Tukey's test calculated using SigmaStat software.

##### Transferrin Recycling Assay

Neurons were incubated with Alexa Fluor 488 Transferrin (10 μg/ml) in serum-free Neurobasal media for 30 min at 37 °C to reach equilibrium. Cells were then washed with PBS twice, and LTP or OGD protocols were performed as described above. After the indicated times, cells were washed twice and processed for immunostaining. Cells transduced with Rab viruses were incubated for 12 to 14 h to allow Rab protein expression before they were used for the recycling experiments. Briefly, neurons were fixed with 2% paraformaldehyde, 4% sucrose in PBS for 20 min and then blocked in 2% serum, 0.02% digitonin for 60 min at room temperature. Cells were then successively incubated with anti GABA_B1_ or GABA_B2_ antibodies overnight at 4 °C and with Cy3-conjugated secondary antibodies for 30 min at room temperature. Confocal fluorescence images from the Alexa Fluor 488, and Cy3 channels were recorded as a series of Z stacks using a Zeiss LSM 510 confocal laser-scanning station with an oil immersion 63 × 1.4 numerical aperture objective (Zeiss). Three-dimensional volumes of z stacks (0.25 μm spacing between single confocal slices) were analyzed using image processing and analysis in Java (ImageJ). The degree of co-localization was assessed in whole cell volumes and sub-volumes by calculating the Pearson's correlation coefficient in the region of interest using a semi-automated algorithm embedded in the JaCoP plugin of ImageJ software (31). The co-localization plugin also performed a two-step analysis to calculate the Pearson's correlation coefficient for the original data and for a large set (∼1000) of images randomized with a grain size determined by the point spread function of the microscope objective. If the Pearson's correlation coefficient of the original image was not greater than 95% of the randomized images, then the co-localization analysis did not continue. In addition, user bias in setting analysis parameters was avoided by using an automated thresholding procedure (31). Histograms presenting the mean correlation coefficient (derived from 19 to 31 cells assessed per treatment condition) are shown with S.D. bars in all figures. Tests of statistical significance for differences between pairwise combinations were calculated using the two-tailed Student's *t* test.

##### Immunoblotting

Proteins were blotted onto Immobilon-P membrane (Millipore) and probed with appropriate primary antibodies overnight after blocking with 5% low-fat milk in TBST (32). For detection, the membrane was incubated with HRP-conjugated secondary antibodies (Sigma, 1:10,000 dilution) for 60 min followed by substrate incubation with BM chemiluminescence blotting substrate (POD) (Roche Applied Science) or SuperSignal West Femto (Pierce). The chemiluminescence signal was detected on Hyperfilm HP (Amersham Biosciences).

## RESULTS

### 

#### 

##### Surface Expression of GABA_B1_ and GABA_B2_ Subunits

**FIGURE 1.**
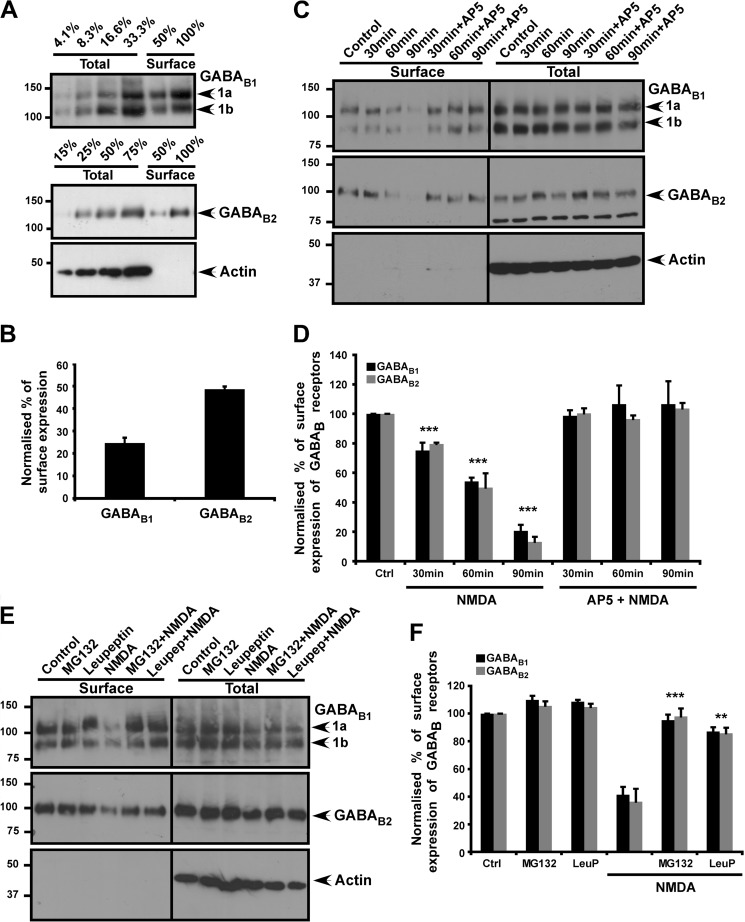
**Bath application of NMDA decreases surface GABA_B_Rs.** *A*, GABA_B1_ and GABA_B2_ surface expression was assessed by surface biotinylation. The total and cell surface GABA_B_ was determined as described under “Experimental Procedures.” *Lane numbers* refer to the amount/percentage of protein loaded compared with 100% of the total protein: GABA_B1_ L1, 4.1%; L2, 8.3%; L3, 16.6%; L4, 33.3%; L5, 50%; and L6, 100%; GABA_B2_ L1, 15%; L2, 25%; L3, 50%; L4, 75%; L5, 50%; and L6, 100%. Blots were probed with anti- GABA_B_ antibodies and reprobed with anti-β-actin antibody to ensure equal loading and the specificity of surface biotinylation. Anti- GABA_B1_ antibody from Santa Cruz Biotechnology was used for blotting, and both the GABA_B1a_ and GABA_B1b_ isoforms were included in the analysis. *B*, quantification of GABA_B1_ and GABA_B2_ protein surface expression ratio (surface to total) measured by biotinylation assays illustrated in *A*. The results shown are the ratios of three independent experiments (*n* = 3). *C*, effect of NMDA on GABA_B1_/GABA_B2_ complex surface expression assessed by surface biotinylation. Cortical neurons were treated with 50 μm NMDA or 50 μm NMDA + 50 μm AP5 for 5 min and then incubated for the times indicated without any drugs. *D*, quantification of the effects of NMDA on GABA_B1_ and GABA_B2_ protein surface expression ratio (surface to total) measured by biotinylation assays illustrated in *C*. The results shown are the ratios of three independent experiments (*n* = 3). ***, *p* ≤ 0.005 compared with control (Students's *t* test). *E*, effects of the proteasome inhibitor MG132 and the lysosome inhibitor leupeptin (*LeuP*) on NMDA-mediated GABA_B1_/GABA_B2_ surface expression. Cultured cortical neurons were treated as in *C* with addition of either 10 μm MG132 or 10 μm leupeptin and incubated for 90 min. *F*, quantification of the effects of MG132 and leupeptin inhibitors on GABA_B1_ and GABA_B2_ protein surface expression ratio (surface to total) measured by biotinylation assays as illustrated in *E*. The results shown are the ratios of four independent experiments (*n* = 4). **, *p* ≤ 0.01; ***, *p* ≤ 0.005 compared with control (*Ctrl*; Student's *t* test).

The steady-state expression of endogenous GABA_B1_/GABA_B2_ subunits in cultured cortical neurones (15–20 days *in vitro*) was determined by surface biotinylation. Under resting conditions 24.59% ± 2.45 of GABA_B1_ and 49.6% ± 1.19 of GABA_B2_ is surface-expressed (Fig. 1, *A* and *B*). The anti-GABA_B1_ antibody recognizes both GABA_B1a_ and GABA_B1b_ subunit isoforms, and both are included in the quantification. Consistent with previous reports (19, 33), these results indicate that there is a larger pool of intracellular GABA_B1_ than GABA_B2_ and infer that the two subunits are regulated by distinct trafficking pathways.

##### Global Activation of Extrasynaptic NMDARs Causes GABA_B_R Internalization

Bath application of glutamate (10, 21) or NMDA (22, 23) has been reported to decrease GABA_B_R surface expression. We recapitulated those results by treating neurons with 50 μm NMDA ± 50 μm AP5 (NMDAR antagonist) for 5 min, allowing the neurons to recover for 30, 60, or 90 min and then measuring surface GABA_B1_ and GABA_B2_. The amount of surface expressed GABA_B1_ and GABA_B2_ was decreased after 30 min and by 90 min after NMDAR activation, and surface GABA_B1_ and GABA_B2_ were levels were decreased by 79.3% ± 4 and 86.8% ± 3.3, respectively (Fig. 1, *C* and *D*). Furthermore, we demonstrate that both the proteasome inhibitor MG132 and the protease inhibitor leupeptin effectively inhibit NMDAR-induced GABA_B_R loss. These data indicate that NMDAR activation causes GABA_B_R degradation (Fig. 1, *E* and *F*) and confirm that the stimulation protocols and trafficking event observed in our cultures are directly comparable with previous reports using glutamate as the agonist (10, 21).

##### Selective Activation of Synaptic NMDARs Promotes GABA_B_R Surface Expression

**FIGURE 2.**
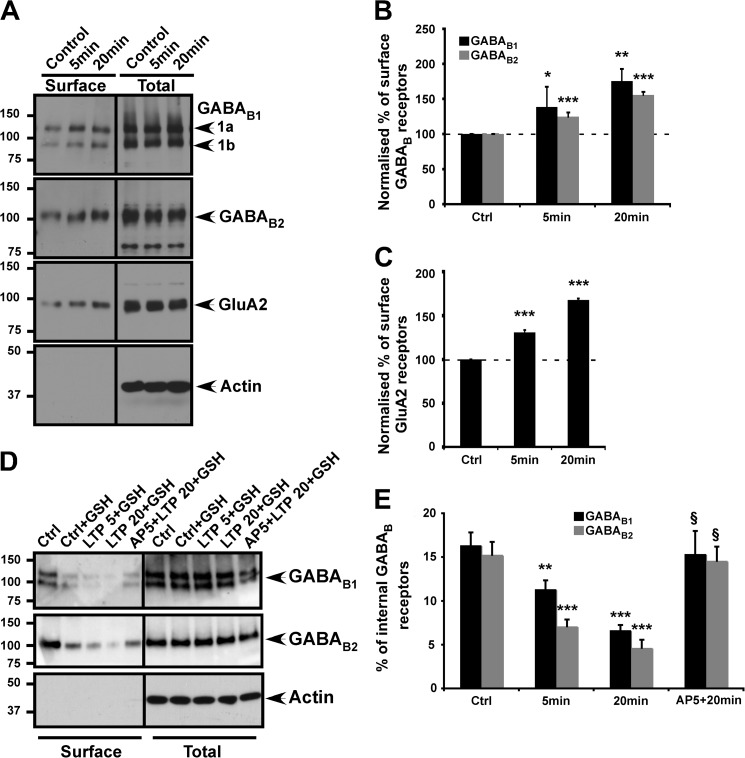
**Activation of synaptic NMDARs increases surface expression of GABA_B_Rs.** *A*, effects of glycine on GABA_B1_, GABA_B2_, and the AMPAR subunit GluA2 assessed by surface biotinylation. Cultured cortical neurons were treated with 200 μm glycine for 3 min and then incubated for 5 or 20 min without glycine before surface biotinylation and harvesting. Blots were probed with anti-GABA_B_ and anti-GluA2 antibodies and reprobed with anti-β-actin antibody to ensure equal loading and the specificity of surface biotinylation. *B*, quantification of the effect of chemLTP on GABA_B1_ and GABA_B2_. *C*, validation of chemLTP showing increased GluA2 protein surface expression. The results shown are the ratios of at least three independent experiments (*n* = 3). **, *p* ≤ 0.01 and ***, *p* ≤ 0.005 compared with control (Student's *t* test). *D*, GABA_B_R endocytosis and recycling was measured by the loss of internalized GABA_B_R specifically labeled with cleavable (S = S linked) biotin. Cortical cultures were surface-biotinylated as described above, and cells were activated by chemLTP protocol and incubated for 5 or 20 min to allow internalized receptors to recycle before cleavage of surface biotin. Residual biotinylated (internal) receptors were then isolated from cells by streptavidin pulldown, and GABA_B_R subunits were detected by Western blotting. Blots were probed with anti- GABA_B1_ and anti-GABA_B2_ antibodies and reprobed with anti-β-actin antibody to ensure equal loading and the specificity of biotinylation. *E*, quantification of the effect of chemLTP on GABA_B1_ and GABA_B2_ rate of disappearance of biotinylated GABA_B_Rs provides a measure of receptor recycling. Leupeptin was included throughout the treatments to block any protein degradation or loss of internalized receptors. The results shown are the ratios of at least three independent experiments (*n* = 3). **, *p* ≤ 0.01 and ***, *p* ≤ 0.005 compared with control (Student's *t* test). § (*p* ≤ 0.005), significant differences between with and without AP5 treatments.

Synaptic and extrasynaptic NMDARs play different roles in neuronal signaling. Activation of synaptic NMDARs mediates synaptic plasticity, whereas activation of extrasynaptic NMDARs leads to excitoxicity (34). We therefore used an extensively characterized chemLTP protocol in which neurons were treated with the NMDAR co-agonist glycine (200 μm, 3 min, 37 °C) (25, 26). This procedure selectively activates only synaptic NMDARs. Strychnine was included in these experiments to block any possible direct effects on glycine receptors. As expected, surface expression of GluA2, was significantly increased using this chemLTP procedure (Fig. 2, *A* and *C*) (25). In direct contrast to the reduction of GABA_B_Rs following bath application of NMDA, chemLTP increased surface expression of both GABA_B1_ and GABA_B2_ (Fig. 2*A*). This increase was both rapid and sustained with 38.3% ± 28 and 24.6% ± 5.9 more GABA_B1_ and GABA_B2_, respectively, at 5 min and 75.4% ± 17.2 and 55.7% ± 4.3 at 20 min after stimulation (Fig. 2*B*). The similar profiles for GABA_B1_ and GABA_B2_ suggest that, under these circumstances, the subunits are likely to be trafficked together as assembled GABA_B_Rs. The peak of AMPAR surface expression following chemLTP occurs between 15 to 20 min after the chemLTP stimulus (25). We therefore routinely monitored changes at 20 min, but we have also monitored experiments for 40 min with similar results.

We also used membrane-impermeant cleavable biotin to label surface-expressed GABA_B_ subunits. After labeling, neurons were incubated at 37 °C to allow constitutive internalization. The neurons containing internalized biotin-labeled GABA_B_ subunits were then subjected to chemLTP, and the surface biotin cleaved at the end of stimulation paradigm. In agreement with the imaging data, the amounts of biotinylated GABA_B_ subunits were decreased after chemLTP, suggesting either a decreased endocytosis or enhanced GABA_B_R recycling (Fig. 2, *D* and *E*). Consistent with a previous report under basal conditions, 15.2% ± 1.5 GABA_B1_ and 16.3% ± 1.46 GABA_B2_ were present inside the cell (internal fraction) (35). Induction of chemLTP decreased the internal fraction of GABA_B1_ to 4.6% ± 1.6 and GABA_B2_ to 6.6% ± 2.6 after 20 min. This effect was blocked by application of the NMDAR antagonist AP5 during chemLTP protocol (Fig. 2, *D* and *E*). Leupeptin was included in all the buffers to prevent degradation during the experiment.

##### ChemLTP Increases GABA_B_R Recycling

**FIGURE 3.**
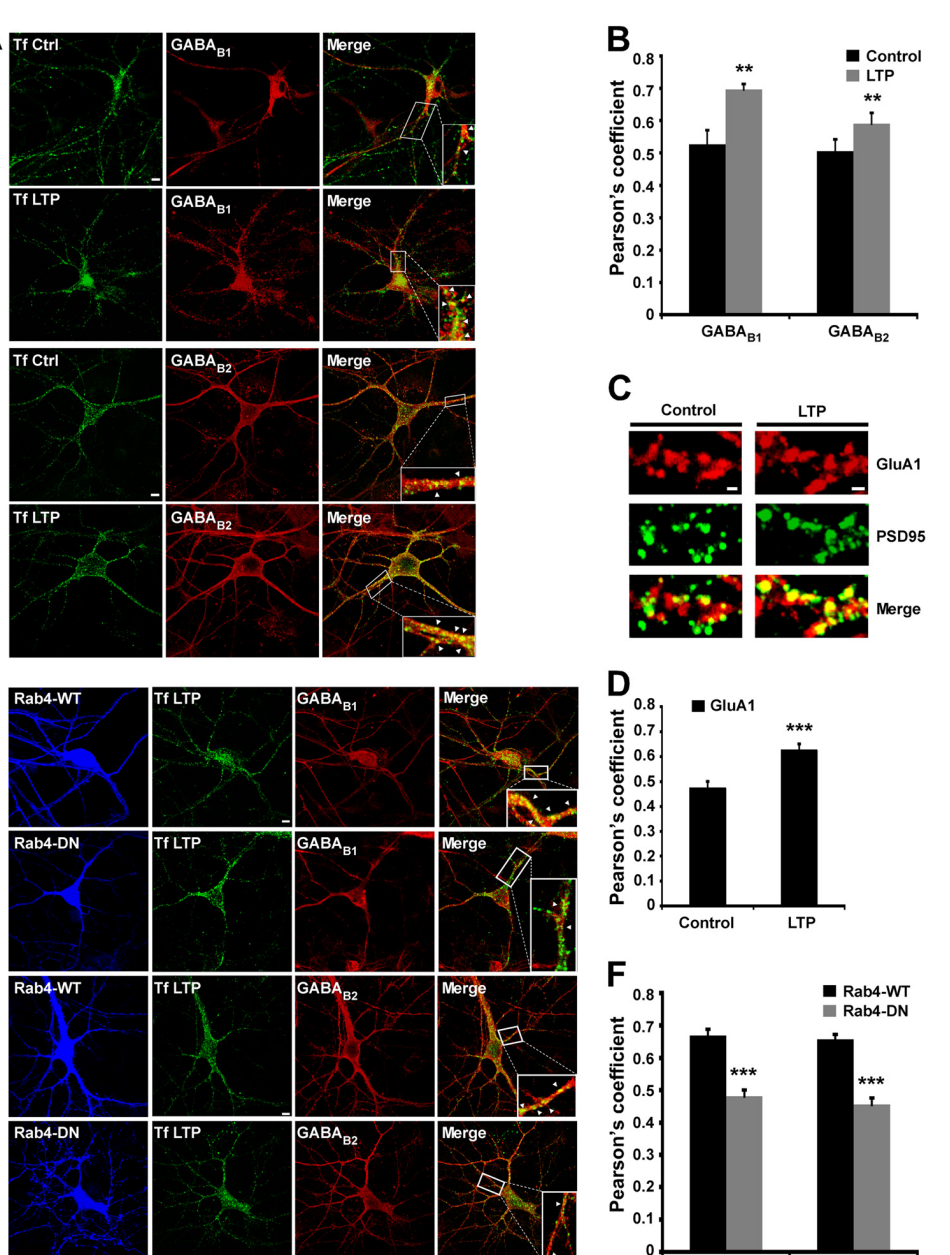
**ChemLTP increases GABA_B_R recycling.** *A*, representative images showing co-localization of GABA_B1_ and GABA_B2_ with the recycling marker Alexa Fluor 488-conjugated transferrin (*Alexa 488-Tf*). Hippocampal neurons were treated with 200 μm glycine for 3 min and incubated at 37 °C for 20 min after replacing the buffer. *B*, quantification of the Pearson's coefficient for the co-localization of GABA_B1_ and GABA_B2_ with Alexa Fluor 488-Tf under basal conditions and 20 min after LTP induction as described in *A*. **, *p* ≤ 0.01 (*n* = 12–16 cells). *C*, immunochemistry showing the co-localization of surface GluA1 (labeled under non-permeabilized conditions) and PSD95 in control and cells treated with glycine-mediated chemLTP as in *A. D*, histograms showing Pearsońs coefficient for the colocalization of GluA1 and PSD95 under basal conditions and after LTP induction as described in *C*. ***, *p* ≤ 0.001 (*n* = 32–33 cells). *E*, WT and dominant-negative (*DN*) Rab4 proteins fused to RFP were expressed in neurons using Sindbis virus and co-localized with GABA_B1_, GABA_B2_, and Alexa Fluor 488-Tf. *F*, Pearson's coefficients for the co-localization of GABA_B_Rs with Alexa Fluor 488-Tf in Rab4-WT and Rab4-DN-transduced cells after LTP induction as *E* (*n* = 19–33 cells). *Arrows* indicate colocalization.

Recycling endosomes supply the AMPARs required for LTP (26). We therefore tested whether a similar recycling mechanism underlies the increase in surface GABA_B_R after chemLTP. We compared GABA_B_R to transferrin receptors (TfRs), which are constitutively internalized into early endosomes and then sorted to recycling endosomes (Fig. 3*A*). As reported previously (36), after a 30-min incubation with Alexa Fluor 488-conjugated transferrin (Tf)-labeled TfR localized in intracellular endosomes. Under basal conditions, the Pearson's coefficients for co-localization of GABA_B1_ and GABA_B2_ with Tf were 0.52 ± 0.04 and 0.50 ± 0.01, respectively, suggesting constitutive recycling occurs for both subunits. Co-localization with Tf was significantly increased after chemLTP with the Pearson's coefficients of 0.69 ± 0.03 for GABA_B1_ and 0.59 ± 0.03 for GABA_B2_, indicating enhanced GABA_B_R recycling (Fig. 3*B*). We used surface staining of the AMPAR subunit GluA1 colocalized with the postsynaptic marker PSD95 as a control for chemLTP protocol in hippocampal neurons. As expected, there was increase in colocalization of GluA1 with PSD95 in neurons subjected to chemLTP (Pearson's coefficients of 0.47 ± 0.02 in controls increasing to 0.62 ± 0.02 in chemLTP), confirming increased AMPAR surface expression and validating the chemLTP protocol (Fig. 3, *C* and *D*).

Rab4 is involved in the rapid receptor recycling (37) and co-localizes with GABA_B_Rs (38). We therefore tested the affects of overexpressing dominant negative Rab4-DN on co-localization of GABA_B1_ and GABA_B2_ with Tf (Fig. 3*E*). In neurons expressing wild type Rab4-WT the increase in the co-localization with Tf after chemLTP was comparable with non-infected control neurons for GABA_B1_ (Pearson's coefficients of GABA_B1_-Tf = 0.67 ± 0.02; GABA_B2_-Tf = 0.65 ± 0.02; Fig. 3*F*). However, in neurons expressing Rab4-DN, there was no increase in GABA_B1_ or GABA_B2_ co-localization with Tf after chemLTP (Pearson's coefficient GABA_B1_-Tf = 0.48 ± 0.02; GABA_B2_-Tf = 0.46 ± 0.02; Fig. 3*F*). We have also validated the Rab4 WT and DN constructs in control conditions. Consistent with previously published results under non-stimulated conditions, no significant changes were observed with the constructs compared with uninfected cells. In neurons expressing Rab4-WT (Pearson's coefficients of GABA_B1_-Tf = 0.57 ± 0.03; GABA_B2_-Tf = 0.55 ± 0.03) and Rab4-DN (Pearson's coefficient GABA_B1_-Tf = 0.53 ± 0.04; GABA_B2_-Tf = 0.52 ± 0.04).

**FIGURE 4.**
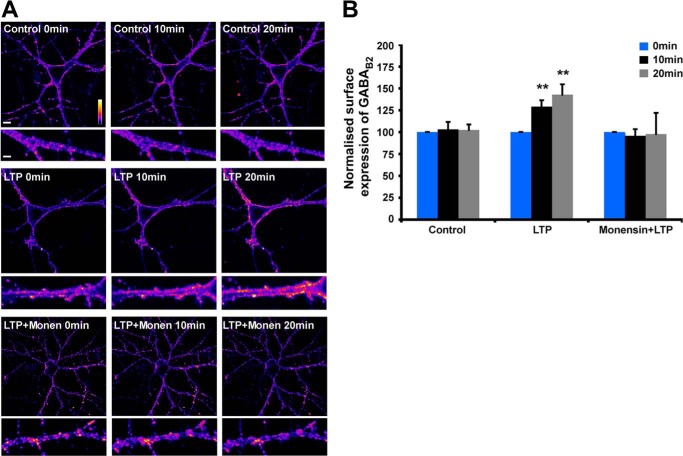
**Monensin prevents chemLTP-induced increases in GABA_B_R surface expression.** *A*, hippocampal neurons were transfected with HA-GABA_B2_ and subjected to the glycine chemLTP protocol in either the presence or absence of 1 μm monensin (*Monen*) for the times indicated. Surface expressed GABA_B_Rs were visualized with HA antibody. *B*, quantification of surface expression of HA-GABA_B2_ containing receptors at 0, 10, and 20 min after LTP induction as described in *A*. **, *p* ≤ 0.01 (*n* = 4–5 cells per condition).

To further investigate the chemLTP-induced increase in surface GABA_B1_ and GABA_B2_, we used live-cell imaging in neurons expressing myc-GABA_B1_ or HA-GABA_B2_. However, consistent with the fact that GABA_B1_ is only weakly surface-expressed in the absence of GABA_B2_, analysis of Myc-GABA_B1_ was confounded by poor surface expression (Fig. 1*B*). Nonetheless, clear increases were observed for HA-GABA_B2_ at 10 and 20 min after chemLTP, and these were prevented by the recycling inhibitor monensin (Fig. 4, *A* and *B*) (39). Taken together, these data strongly support the proposal that NMDAR activation controls GABA_B_R surface expression via regulation of the recycling endosomal pathway.

##### OGD Differentially Affects Surface Expression of GABA_B_R Subunits

**FIGURE 5.**
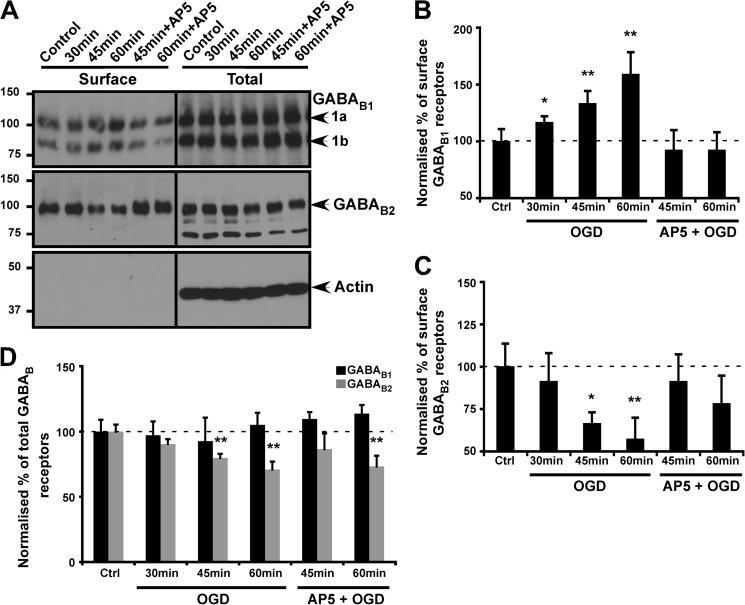
**OGD increases GABA_B1_ and decreases GABA_B2_ surface expression.** *A*, cultured cortical neurons were exposed to OGD for the times indicated, and total and cell surface GABA_B1_ and GABA_B2_ were determined as described under “Experimental Procedures.” Because there were OGD-induced changes in both total and surface expression of receptors, the fractions are normalized to actin levels individually, and the normalized percentages for surface (*B* and *C*) and total (*D*) are plotted separately. Blots were probed with anti-GABA_B_ antibodies and reprobed with anti-β-actin antibody to ensure equal loading and the specificity of surface biotinylation. *B* and *C*, quantification of surface GABA_B1_ and GABA_B2_, respectively, measured by biotinylation assays as illustrated in *A* (surface to actin ratio). *D*, total protein levels of GABA_B1_ and GABA_B2_ (total to actin). The results shown are the ratios of three independent experiments (*n* = 3). *, *p* ≤ 0.05 and **, *p* ≤ 0.01 compared with control (*Ctrl*; Students's *t* test). The *dashed line* represents control levels − 100%.

Our results demonstrate that bath application of NMDA causes GABA_B_R internalization, whereas chemLTP evoked by activation of synaptic NMDARs increases surface GABA_B_R expression. Therefore, we next investigated how excitotoxic activation of NMDARs during OGD affects GABA_B_R surface expression. We have shown previously that 24 h after exposure of rat organotypic hippocampal slice cultures to OGD (45 min), there is a marked decrease in the total levels of GABA_B2_ (∼75%) but no significant change in the levels of GABA_B1_ (19). However, due to technical considerations, the slice culture experiments did not assess levels of GABA_B_R surface expression. Here, we have assessed the effects of 30, 45, and 60 min OGD on surface expression and total levels of GABA_B_R subunits in dispersed cultured neurons (Fig. 5*A*). Consistent with our slice data, in this dispersed cell culture system total levels of GABA_B2_ were decreased (29.1% ± 15) and total levels of GABA_B1_ were unchanged (Fig. 5, *A* and *D*). Interestingly, however, OGD significantly increased surface levels of GABA_B1_ (59.2% ± 18.9), whereas GABA_B2_ surface expression was decreased by 42.3% ± 12.5 (Fig. 5, *A–C*). The NMDAR antagonist AP5 blocked these changes in GABA_B1_ and GABA_B2_ surface expression to differing extents. Thus, OGD regulates the surface trafficking of GABA_B_R subunits in opposite directions.

**FIGURE 6.**
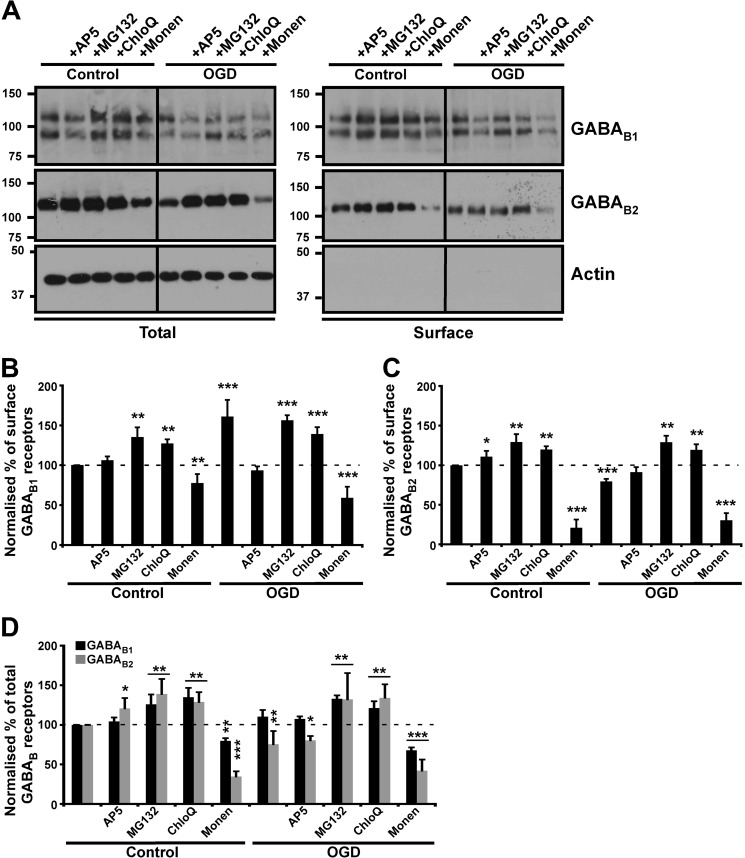
**GABA_B_R trafficking in OGD.** Effects of OGD on GABA_B1_ and GABA_B2_ surface expression assessed by surface biotinylation. *A*, cultured cortical neurons were incubated with 50 μm AP5 (NMDAR antagonist), 10 μm MG132 (proteasome inhibitor), 10 μm chloroquine (*ChloQ*, lysosomal inhibitor) or 1 μm monensin (*Monen*, recycling blocker). Blots were probed with anti-GABA_B_ antibodies and reprobed with anti-β-actin antibody to ensure equal loading and the specificity of surface biotinylation. *B* and *C*, quantification of surface expression for GABA_B1_ and GABA_B2_, respectively (surface to actin ratio). *D*, total protein levels for GABA_B1_ and GABA_B2_. The data are the ratios of three independent experiments (*n* = 3). *, *p* ≤ 0.05; **, *p* ≤ 0.01; ***, *p* ≤ 0.005, compared with OGD-treated neurons (Students's *t* test). The *dashed line* is control levels − 100%.

Protein degradation plays a pivotal role in receptor stability, trafficking, and recycling (40). The lysosomal inhibitor chloroquine and the proteasomal inhibitor MG132 increase total levels of GABA_B1_ and GABA_B2_ under both control and OGD conditions (Fig. 6, *A* and *D*). Furthermore, OGD-induced GABA_B1_ surface expression was enhanced by either chloroquine or MG132, indicating that both proteasomal and lysosomal pathways can degrade GABA_B1_ (Fig. 6, *A* and *B*). Intriguingly, chloroquine or MG132 also prevented the OGD-induced decrease in surface GABA_B2_ (Fig. 6, *A* and *C*). In contrast, inclusion of the recycling inhibitor monensin during OGD caused a marked decrease in total levels and surface expression levels of GABA_B_Rs (Fig. 6, *A–D*). We attribute this to blockade of recycling leading to the sorting of constitutively endocytosed receptors to degradation pathways during OGD (11). We also observed that in control conditions there was decrease in surface and total levels of GABA_B_Rs following monensin treatment. This observation is consistent with previously published data suggesting that block in recycling traffics the receptors to lysosomes (35). Similar to our chemLTP experiments where monensin prevented the increase in GABA_B_ receptors, the fact that monensin blocked the OGD-induced increase in surface GABA_B1_ suggests that enhanced recycling is a core mechanism underlying these effects.

##### GABA_B1_ and GABA_B2_ Recycling Are Separately Regulated during OGD

**FIGURE 7.**
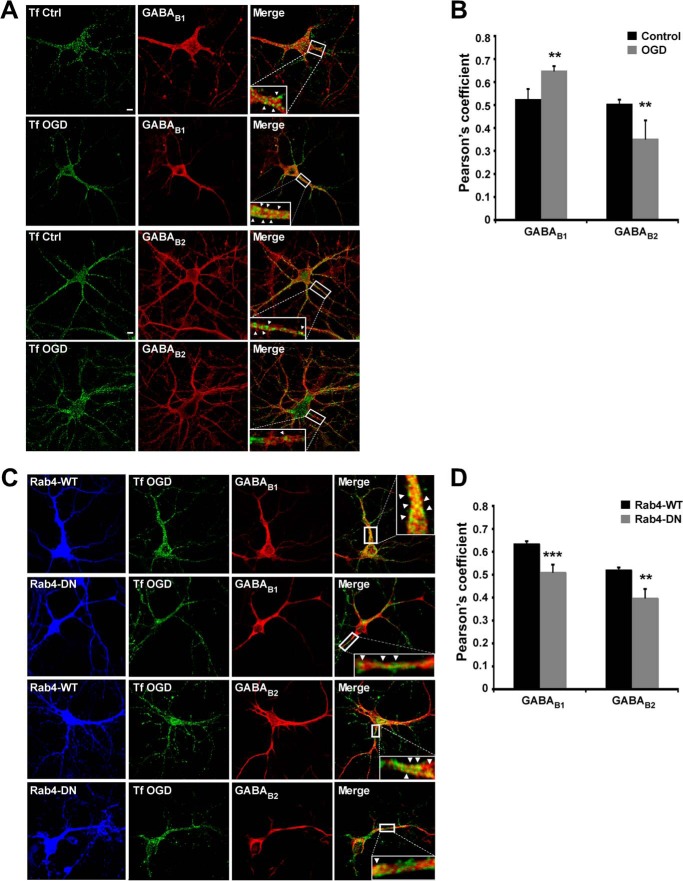
**OGD regulates GABA_B_R expression by modulating recycling.** *A*, co-localization of GABA_B1_ and GABA_B2_ with Alexa Fluor 488-Tf in control (*Ctrl*) and OGD-treated cultured hippocampal neurons. *B*, Pearson's coefficients for the co-localization of GABA_B1_ and GABA_B2_ with Alexa Fluor 488-Tf. The data are representative of at least three separate experiments (*n* = 22 cells per condition). **, *p* ≤ 0.01. *C*, co-localization of GABA_B1_ and GABA_B2_ with Alexa Fluor 488-Tf in control and OGD-treated cultured hippocampal neurons expressing Rab4-WT or Rab4-DN. *D*, Pearson's coefficients for the co-localization of GABA_B1_ and GABA_B2_ with Alexa Fluor 488-Tf. The data are the ratios of independent experiments (*n* = 2, 20–21 cells per condition). **, *p* ≤ 0.01; ***, *p* ≤ 0.005, compared with OGD-treated neurons (Students's *t* test). *Arrows* indicate colocalization.

To investigate the recycling properties of GABA_B_R subunits following OGD, we performed functional co-localization analysis with the TfR. As shown in Fig. 3, under basal conditions, the Pearson's coefficients of GABA_B1_ and GABA_B2_ co-localization with TfR were 0.52 ± 0.03 and 0.51 ± 0.02, respectively, consistent with both subunits undergoing constitutive recycling. After 30 min of OGD, however, Pearson's coefficients for co-localization with TfR were 0.65 ± 0.01 and 0.35 ± 0.07 for GABA_B1_ and GABA_B2_, respectively (Fig. 7, *A* and *B*). We next tested the effects of expressing Rab4-WT or Rab4-DN (Fig. 7*C*). In Rab4-WT expressing neurons, the increase in the co-localization with Tf after OGD was similar to non-infected cells for GABA_B1_ (Fig. 7, *A* and *B*). For GABA_B2_, however, the levels were comparable to control (Pearson's coefficient GABA_B1_-Tf = 0.63 ± 0.02; GABA_B2_-Tf = 0.51 ± 0.03) (Fig. 7, *C* and *D*). This suggests that Rab4-WT overcomes the OGD-induced reduction on GABA_B2_ recycling. Expression of Rab4-DN blocked OGD-induced changes in GABA_B_R subunit co-localization with Tf (Pearson's coefficient GABA_B1_-Tf = 0.51 ± 0.03; GABA_B2_-Tf = 0.40 ± 0.03) (Fig. 7*D*). Interestingly, expressing Rab4-DN did not alter OGD-induced changes in GABA_B2_, suggesting that there is no further decrease in recycling rates during OGD when Rab4-DN is expressed. Consistent with our biochemistry data, these results demonstrate differential trafficking of the individual subunits and indicate that the increase in GABA_B1_ surface expression is due to increased recycling and that the decrease in GABA_B2_ surface expression is due to reduced recycling and/or increased sorting to degradative pathways.

## DISCUSSION

**FIGURE 8.**
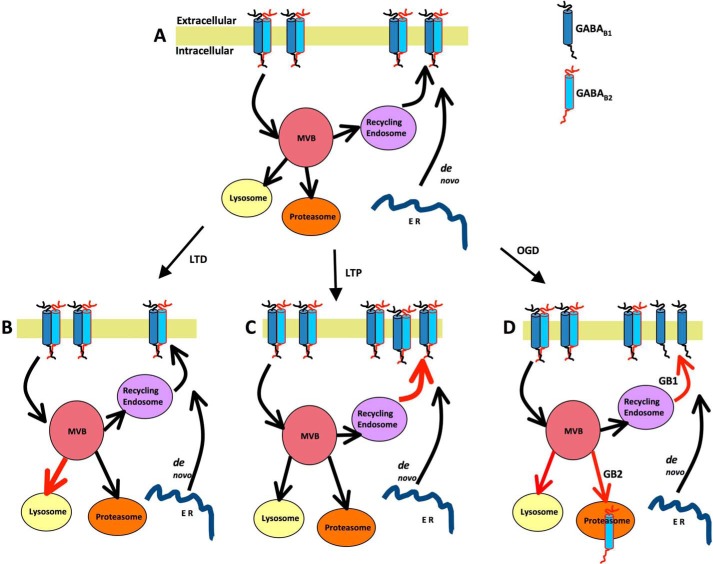
**Schematic of chem-LTD, chemLTP, and OGD-mediated GABA_B_R trafficking.** *A*, under basal state, GABA_B_Rs are cycled constitutively at plasma membrane. *B*, after LTD, endocytosed GABA_B1_ and GABA_B2_ are sorted to lysosomes (21, 22). *C*, after chemLTP, endocytosed GABA_B1_ and GABA_B2_ are sorted to recycling endosomes and returned to the plasma membrane increasing surface expression of both GABA_B_ subunits. *D*, OGD leads to differential trafficking of GABA_B_Rs. There is enhanced recycling of GABA_B1_ to plasma membrane, whereas GABA_B2_ is degraded via proteasomal and/or lysosomal pathways. *MVB*, multivesicular bodies; *ER*, endoplamic reticulum.

We show that activation of synaptic NMDARs increases GABA_B_R surface expression by enhancing recycling of both GABA_B1_ and GABA_B2_ (Fig. 8). We used well established and routinely used glycine-induced chemLTP (25, 26, 41, 42). The co-agonist glycine (43) acts synergistically with spontaneously released glutamate in the synaptic cleft to activate synaptic NMDARs and induce LTP. To exclude any potential confounding effect from activation of inhibitory glycine receptors, we included the glycine receptor antagonist strychnine in the LTP buffer.

Our results show that, like excitatory AMPARs, inhibitory GABA_B_Rs undergo a form of NMDAR-dependent plasticity. Furthermore, these data are consistent with the observation that NMDAR-invoked AMPAR mediated LTP in postsynaptic CA1 pyramidal neurons also causes LTP of the slow inhibitory postsynaptic current-mediated by GABA_B_Rs (44).

GABA released from interneurons can activate both pre- and postsynaptic GABA_B_Rs at glutamatergic synapses (45). Enhanced presynaptic GABA_B_R surface expression reduces glutamate release and elicits hyperpolarizing inhibitory postsynaptic potentials that facilitate the Mg^2+^ block of NMDARs and reduce Ca^2+^ signaling (17, 18, 20). Thus, the likely role of the chemLTP-induced increase GABA_B_Rs at glutamatergic synapses is to enhance inhibitory tone to counterbalance the increase in AMPARs and prevent hyperexcitability. These data highlight the complex inter-relationship and cross-talk between inhibitory and excitatory receptors that varies depending on the profile of NMDAR activation.

Under basal conditions, both GABA_B1_ and GABA_B2_ co-localize with TfR-positive recycling endosomal compartments indicating that GABA_B_Rs undergo constitutive recycling. Furthermore, this co-localization increases following chemLTP and the chemLTP-induced increase in GABA_B1_ and GABA_B2_ surface expression is prevented by blocking recycling with monensin or Rab4-DN. These results suggest that selective activation of synaptic NMDARs enhances sorting to recycling pathways and away from degradation. Moreover, they extend previous observations that heterodimeric GABA_B_Rs undergo clathrin- and dynamin-1-dependent endocytosis and recycle back to the cell surface (21). As this is routine practice in the glutamate and GABA receptor fields, we used hippocampal neurons for immunocytochemistry and cortical neurons for biochemistry. Importantly, in this study, and the many previous studies, the biochemical data from cortical neurons is entirely consistent with the imaging data from hippocampal neurons.

Consistent with previous observations (10, 21–23), we found that NMDA (5 min, 50 μm) decreased surface expression of both GABA_B1_ and GABA_B2_. Activation of extrasynaptic NMDARs is neurotoxic and can serve as a potent signal for cell death (24). We therefore reasoned that similar effects on GABA_B_R trafficking might occur following excitotoxic events similar to epilepsy or in severe oxidative stress such as ischemia where there is prolonged and diffuse glutamate release.

Our previous work demonstrated that OGD causes a 70% reduction in total levels of GABA_B2_ but has no effect on total levels of GABA_B1_ (19). Here, we confirm that total levels of GABA_B2_ are decreased and GABA_B1_ totals are unchanged. Additionally, we show that pathological activation of NMDARs during OGD results in a 50% increase in surface-expressed GABA_B1_ and a 40% decrease in surface GABA_B2_, presumably altering the composition of surface GABA_B_Rs (Fig. 8). OGD is a form of severe metabolic oxidative stress that has multiple effects in cells, including raised free radical production, ATP depletion, increased levels of intracellular Ca^2+^, and the release of high amounts of glutamate. Under these stressed conditions, the individual GABA_B_R subunits appear to undergo independent endocytosis, recycling, and degradation. These results highlight the fact that exogenous agonist addition does not necessarily have the same effects as physiological or pathophysiological stimulation.

Taken together, we interpret our data to suggest that surface expressed GABA_B_R complexes are dynamic and may disassemble while in the membrane. Interestingly, chronic stimulation with capsaicin can cause GABA_B_R heterodimers to dissociate with the individual subunits subject to differential trafficking (12). Indeed, a high proportion of GABA_B_R subunits are monomers segregated in distinct dendritic compartments, whereas assembled GABA_B_R heteromers are preferentially located at the plasma membrane (46). How the differential regulation of GABA_B_R subunits is achieved, and the consequent cellular effects, are important outstanding questions.

We attribute the increase in surface GABA_B1_ following OGD to the recycling of a higher proportion of this subunit to the membrane because the effect was blocked by monensin. In addition, OGD directly promotes GABA_B2_ degradation following internalisation and the OGD-induced decrease in surface GABA_B2_ was completely blocked by proteasome or lysosome inhibition. Because functional GABA_B_Rs have to be heterodimers, the decrease in surface GABA_B2_ causes a substantial decrease in GABA_B_R signaling following OGD. Further work is needed, but we hypothesize that this loss of inhibition could provide a mechanism to drive damaged cells toward death via excitoxicity. In addition, the increase in GABA_B1_ subunit surface expression might act to chelate GABA or heterodimerise with other G protein-coupled receptors (47) to produce differential signaling.

In conclusion, our findings indicate that the fates of surface expressed GABA_B_R subunits are separately regulated under chemLTP and OGD conditions and that individual subunits recycle independently. Based on these data, we propose that the surface expression or endocytosis of GABA_B_Rs arising from different modes of NMDAR activation represents an important mechanism that regulates neuronal responsiveness and survival.

## Footnotes

6 The abbreviations used are:

GABA: γ-amino butyric acid
NMDA: *N*-methyl d-aspartate
OGD: oxygen-glucose deprivation
LTP: long term potentiation
chemLTP: chemically induced LTP protocol
TfR: transferrin receptor.
